# Loss of Selenium-Binding Protein 1 Decreases Sensitivity to Clastogens and Intracellular Selenium Content in HeLa Cells

**DOI:** 10.1371/journal.pone.0158650

**Published:** 2016-07-12

**Authors:** Changhui Zhao, Huawei Zeng, Ryan T. Y. Wu, Wen-Hsing Cheng

**Affiliations:** 1 Department of Food Quality and Safety, College of Food Science and Engineering, Jilin University, Changchun, Jilin Province, 130062, China; 2 Department of Nutrition and Food Science, University of Maryland, College Park, Maryland 20742, United States of America; 3 United States Department of Agriculture, Agricultural Research Service, Grand Forks Human Nutrition Research Center, Grand Forks, North Dakota 58202, United States of America; 4 Department of Food Science, Nutrition & Health Promotion, Mississippi State University, Mississippi State, Mississippi 39762, United States of America; Taipei Medical University, TAIWAN

## Abstract

Selenium-binding protein 1 (SBP1) is not a selenoprotein but structurally binds selenium. Loss of SBP1 during carcinogenesis usually predicts poor prognosis. Because genome instability is a hallmark of cancer, we hypothesize that SBP1 sequesters cellular selenium and sensitizes cancer cells to DNA-damaging agents. To test this hypothesis, we knocked down SBP1 expression in HeLa cervical cancer cells by employing a short hairpin RNA (shRNA) approach. Reduced sensitivity to hydrogen peroxide, paraquat and camptothecin, reactive oxygen species content, and intracellular retention of selenium after selenomethionine treatment were observed in SBP1 shRNA HeLa cells. Results from Western analyses showed that treatment of HeLa cells with selenomethionine resulted in increased SBP1 protein expression in a dose-dependent manner. Knockdown of SBP1 rendered HeLa cells increased expression of glutathione peroxidase-1 but not glutathione peroxidase-4 protein levels and accelerated migration from a wound. Altogether, SBP1 retains supplemental selenium and sensitizes HeLa cancer cells to clastogens, suggesting a new cancer treatment strategy by sequestering selenium through SBP1.

## Introduction

There are two selenium-binding proteins known to exist [1, 2]. Different from selenoproteins that decode the UGA codon as selenocysteine co-translationally, selenium-binding proteins do not contain an in-frame UGA codon or selenocysteine but post-translationally bind selenium as a ligand. Selenium-binding protein 1 (SBP1) is a 56-kD cytosolic protein and heavily tyrosine-phosphorylated [3]. SBP1 covalently binds selenium possibly via a selenosulfide bond through the domain surrounding cysteine-57 [4, 5].

SBP1 is frequently under-expressed in cancer cells, including those of esophagus [6], lung [7], stomach [8], liver [9], colorectal [10, 11], prostate [12] and ovary [13, 14] origins. Although the pathogenesis is not fully understood, loss of SBP1 usually predicts poor prognosis as demonstrated in lung [7], colon [10, 11], prostate [15], malignant pleural mesothelioma [16] and liver cancer, as well as vascular invasion and recurrence [9]. Nonetheless, it has been shown that SBP1 interacts with glutathione peroxidase-1 (GPX1) and decreases GPX activity [9, 15, 17, 18]. Because GPX1 is a selenoenzyme that decomposes hydrogen peroxide, SBP1 may thus indirectly promote oxidative stress. On the other hand, body selenium status and speciation can modulate carcinogenesis [19], suggesting another plausible target of SBP1 action in carcinogenesis. Selenium compounds in excess can generate cytotoxic effects and induce apoptosis and DNA damage response in cancer cells [20–22]. Based on the selenium-binding feature of SBP1, increased SBP1 protein expression in principle could increase intracellular selenium content and thus cytotoxicity. This implicates SBP1 in the inhibition of carcinogenesis. Therefore, we hypothesized that loss of SBP1 could modulate reactive oxygen species (ROS) and selenium levels and the sensitivity of cancer cells to DNA-damaging agents. This was tested in the SBP1 shRNA and scrambled shRNA HeLa cells we generated.

## Materials and Methods

### Cell culture

HeLa cervical cancer cells (ATCC, Manassas, VA) were cultured in DMEM (Corning cellgro®, Mediatech) supplemented with 10% fetal bovine serum (Sigma), 100 U/ml penicillin and streptomycin (Mediatech) at 37°C in a 5% CO_2_ incubator (Thermo Scientific). The basal medium contains selenium at 0.036 μM by analysis [23].

### SBP1 knockdown

shRNA knockdown was carried out as detailed previously [23]. Briefly, stable shRNA HeLa cells were generated following manufacturer’s instructions (BLOCK-iT™ U6 RNAi Entry Vector kit and BLOCK-iT™ Lentiviral RNAi Expression System, Invitrogen) by using SBP1 shRNA (5'-GGCTTATTCCCTTGGAGATCC) and scrambled shRNA (5’-CCTAAGGTTAAGTCGCCCTCGC) sequences. A stable knockdown clone was selected by incubation with blasticidin for 2 weeks after a single cell was picked and proliferated. Once a clone was generated, blasticidin was no longer used but SBP1 knockdown efficiency was assessed before performing an experiment.

### Clonogenic assay

Clonogenic assays were performed as reported previously [23]. Briefly, hydrogen peroxide, methyl viologen dichloride hydrate (paraquat) and (S)-(+)-camptothecin (all from Sigma) were applied to HeLa cells for 1 day, followed by recovery for 7 days in clastogen-free medium and then incubation with 0.05% crystal violet in 2% ethanol for 20 min. A colony was defined as that containing more than 50 cells visualized under a phase contrast microscope.

### Intracellular ROS levels

Cells were seeded onto glass bottom culture dishes and incubated overnight at 37°C in a 5% CO_2_ incubator. Cells were incubated with 5 μmol/L CM-H2DCFDA (Invitrogen) in a phenol red-free RPMI medium (Mediatech) for 45 min at 37°C in the dark, followed by image acquisition under a Zeiss AxioObserver 100 fluorescence microscope. Fields of cells were randomly taken and quantified using the intensity tool of the software in the Zeiss AxioObserver 100 fluorescence microscope package.

### Selenium analysis

Confluent cells were washed four times with PBS, treated with a lysis buffer containing NaOH (0.2 mol/L) and 0.2% SDS for 15 min, and then scraped into 1.5 mL Eppendorf tubes. After determination of protein concentrations by bicinchoninic acid assay (Thermo Scientific), the samples were subjected to selenium analysis as described previously [24].

### Western analysis

Cell lysates were prepared by using a RIPA buffer containing Sigmafast Protease Inhibitor Cocktail and Phosphatase Inhibitor Cocktail 3 (Sigma). Protein concentrations were quantified by using the bicinchoninic acid assay (Thermo Scientific). Proteins were separated by SDS PAGE with 10% acrylamide, transferred onto PVDF membranes and incubated with antibodies against SBP1 (1: 1000, Abcam), GPX1 and GPX4 (1:1000, GeneTex), and β-tublin (1: 10,000, Santa Cruz) overnight at 4°C. After being washed in a TBST buffer, the membranes were blocked in 5% non-fat milk (Bio-Rad) for 1 h at room temperature. After secondary antibody incubation, protein levels were represented as chemiluminescence signals after incubation with Supersignal Femto solutions (Pierce) and detected by using a ChemiDoc™ MP Imaging System (Bio-Rad). Band intensity was semi-quantified by using WCIF ImageJ 1.37c. The relative values were normalized with that of β-tubulin.

### Wound assay

Cells were seeded onto 6-well plates and cultured until reaching 90% confluent at 37°C in a 5% CO_2_ incubator. The cells were cultured in a complete medium containing only 0.2% FBS for 24 h, scratched straight with a 200 μL pipet tip, washed once with PBS and photographed under a light microscope. The cells were then resumed to be cultured in a complete medium. Pictures were taken at 0, 24 and 72 h to follow migration of the cells. Wound distance was estimated by averaging three randomly chosen horizontal space between the lines.

### Sulforhodamine B proliferation assay

Sulforhodamine B (SRB) is a bright pink aminoxanthene dye with two sulfonic groups capable of binding basic amino acid residues stochastically under mild acidic conditions in cells. Briefly, cells (1.9 x 10^4^/well) were seeded onto 96-well plates in triplicate and grown for 3 h or 72 h. Cells were then fixed with cold 10% TCA at 4°C for 2 h. The plate was washed 4 times with slowly running tap water via plastic tubing and then air-dried at room temperature in a hood. Cells were stained by incubating with 0.057% SRB for 30 min, followed by quickly rinsing the cells 4 times with 1% acetic acid to remove unbound dye. After being air dried in a hood, 200 μl of Tris base solution (pH 10.5, 10 mmol/L) was added and the plate was shaked on a gyratory shaker for 5 min. OD values were measured at 492 nm by a plate reader. Cell proliferation was estimated based on the ratio of OD values at 72 h over those at 3 h.

### Data analysis

Single factor analysis of variance (ANOVA) followed by Student’s *t*-test or two-way ANOVA followed by Tukey’s post-hoc tests were used for comparisons of the means by using IBM SPSS Statistics 21.

## Results

### SBP1 knockdown desensitized HeLa cells to three clastogens

**Fig 1:**
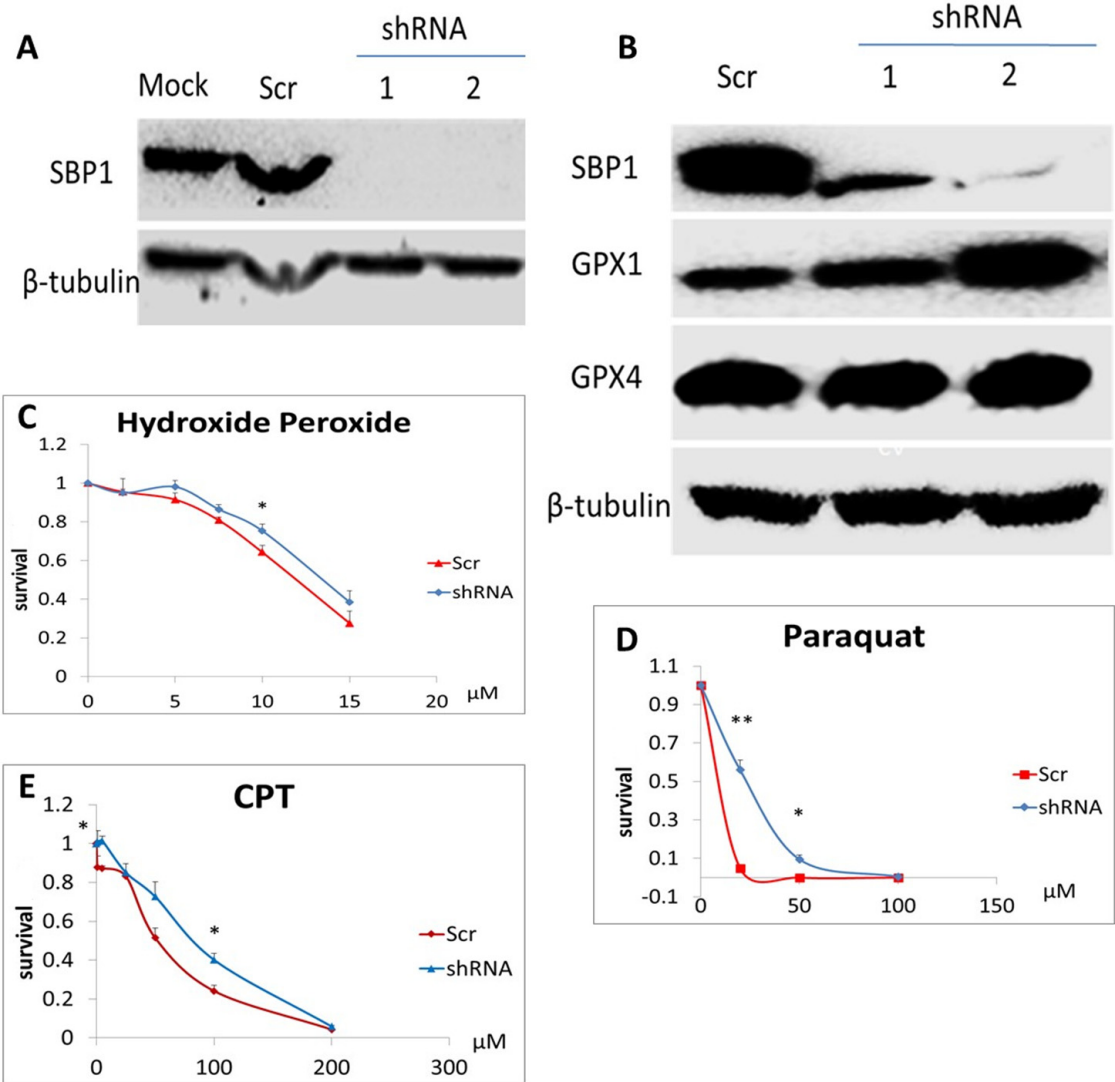
Knockdown of SBP1 desensitized HeLa cells to three clastogens and increased GPX1 protein levels. (A) Efficacy of SBP1 shRNA knockdown in two clones of HeLa cells as evidenced by Western analysis. (B) Effect of SBP1 knockdown on the protein levels of GPX1 and GPX4 by Western analysis. (C-E) Clonogenic assays were employed to determine sensitivity of scrambled and SBP1 shRNA HeLa cells to hydrogen peroxide (2–15 μM, C), paraquat (20–100 μM, D), and camptothecin (CPT, 5–200 μM, E) for 24 h, followed by a 7-day recovery and then crystal violet staining for detection of viable colonies. Values are means ± SEM (n = 3). *, p< 0.05 and **, p< 0.01, compared to scrambled shRNA HeLa cells.

The SBP1 protein expression was effectively suppressed in SBP1 shRNA HeLa cells as compared to mock or scrambled shRNA cells (Fig 1A). Knockdown of SBP1 increased protein levels of GPX1 but not GPX4 in HeLa cells (Fig 1B). Results of clonogenic assays showed that while scrambled shRNA HeLa cells were sensitive to hydrogen peroxide, paraquat, and camptothecin in a dose-dependent manner, SBP1 shRNA HeLa cells showed decreased sensitivity to these three DNA-damaging agents (Fig 1C–1E). Specifically, SBP1 shRNA cells were significantly more resistant (*p* < 0.05) than scrambled shRNA cells to hydrogen peroxide treatment at 10 μM, paraquat at 25 and 50 μM, and camptothecin at 5, 100 and 200 μM.

### SBP1 knockdown decreased ROS levels in HeLa cells

**Fig 2:**
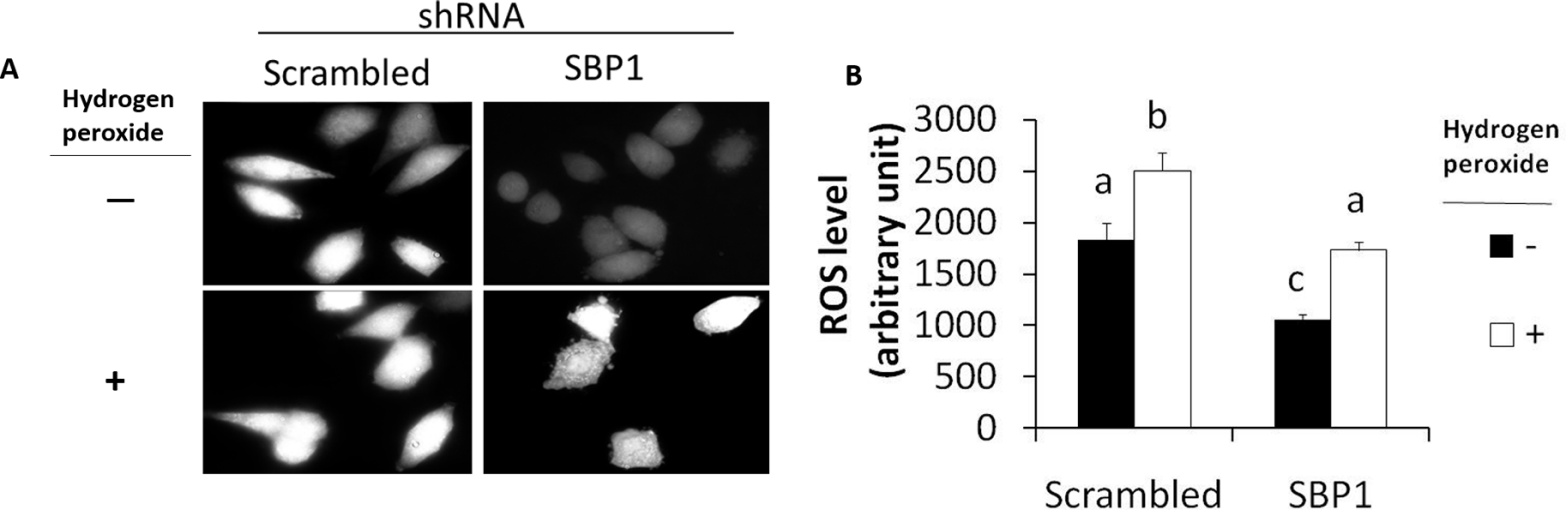
Knockdown of SBP1 decreases ROS levels in HeLa cells before and after treatment with hydrogen peroxide. Scrambled and SBP1 shRNA HeLa cells were cultured in the presence or absence of hydrogen peroxide (100 μM, 3 h), followed by incubation with CM-H2DCFDA (5 μM, 15 min, 37°C) and acquisition of FITC signal through a fluorescence microscope. (A) Representative pictures as observed under a fluorescence microscope. (B) FITC signals were quantitated using the intensity tool of the Zeiss AxioObserver 100 software. Values are means ± SEM (n = 3) and differ when not sharing a common letter (p< 0.05).

While treatment with hydrogen peroxide increased ROS levels in both scrambled and SBP1 shRNA HeLa cells, the latter cells contained less ROS than the former cells in the absence or presence of hydrogen peroxide treatment by 43.7% and 29.0%, respectively (Fig 2A and 2B).

### Decreased intracellular selenium content by SBP1 knockdown and increased SBP1 expression after selenomethionine treatment in HeLa cells

**Fig 3:**
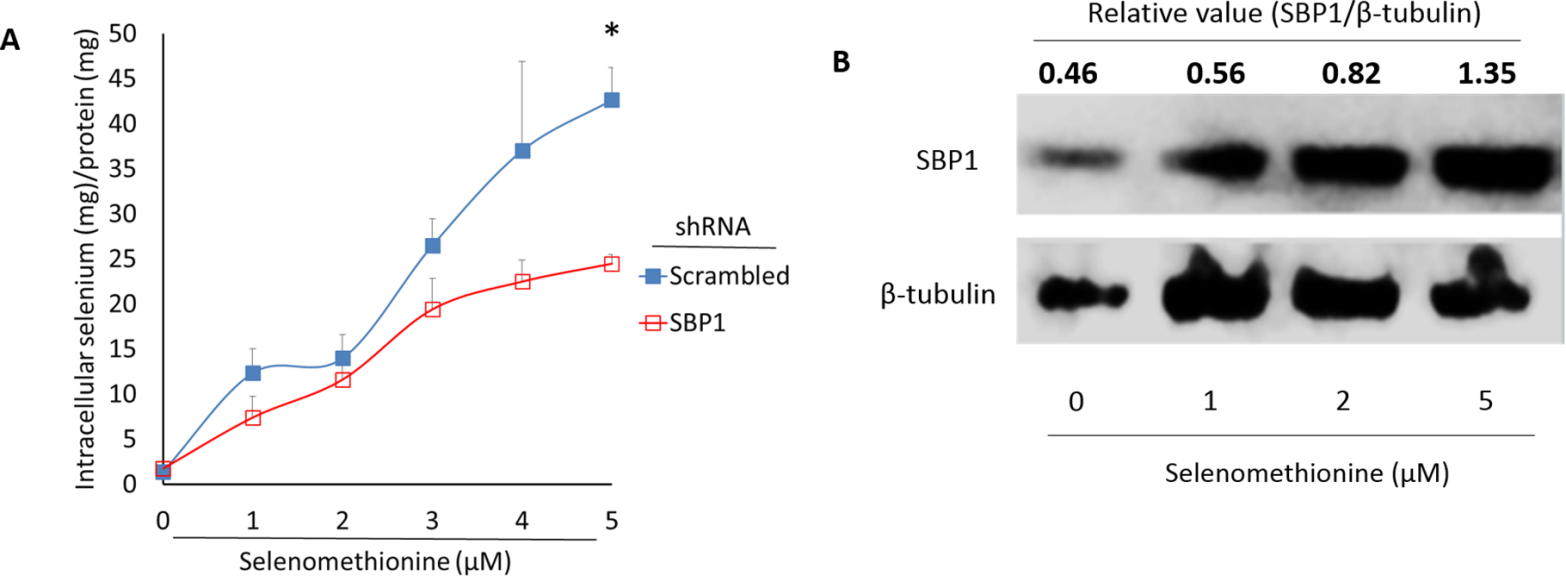
Increased SBP1 protein levels and SBP1-dependent retention of intracellular selenium content after selenomethionine treatment in HeLa cells. (A) Intracellular selenium concentration was determined in scrambled and SBP1 shRNA HeLa cells treated with a gradient concentration of selenomethionine (0–5 μM, 3 h). Values are means ± SEM (n = 3). *, p< 0.05, compared to SBP1 shRNA HeLa cells. (B) Western analyses of SBP1 and β-tubulin in HeLa cells treated with a gradient concentration of selenomethionine (0–5 μM, 24 h).

The absorption efficacy of selenomethionine is greater than three other selenium species (methylselenocysteine, selenate and selenite) and can reach a plateau 3 h after incubation in Caco-2 cells mimicking intestinal absorption [25]. Because selenium concentrations in the plasma of healthy people are estimated to be 1–2 μM and selenium is mainly protein-bound but not freely present in biological samples [26, 27], selenomethionine at concentrations up to 5 μM has been employed. While intracellular selenium concentrations were linearly increased 3 h after incubation with selenomethionine in a dose-dependent manner (1–5 μM), the extent was less in SBP1 shRNA than scrambled shRNA cells (Fig 3A). While intracellular selenium concentration had no discernible change between SBP1 and scrambled shRNA cells when selenomethionine was added at 1–2 μM, differences between these two cells widened as the selenium dose increased, to the extent that there was a 42.6% decrease (p< 0.05) in SBP1 shRNA cells at 5 μM. Because SBP1 could bind selenium covalently [4], we next determined whether treatment of cells with selenomethionine affected SBP1 expression. Results from Western analyses showed that SBP1 protein levels were increased by selenomethionine treatment in a dose-dependentmanner (0–5 μM) in HeLa cells (Fig 3B). Altogether, these results collectively suggest that SBP1 plays important roles in the regulation of cellular selenium status, especially when selenium is supplemented at high doses.

### SBP1 knockdown did not affect cell morphology but promoted migration of HeLa cells

**Fig 4:**
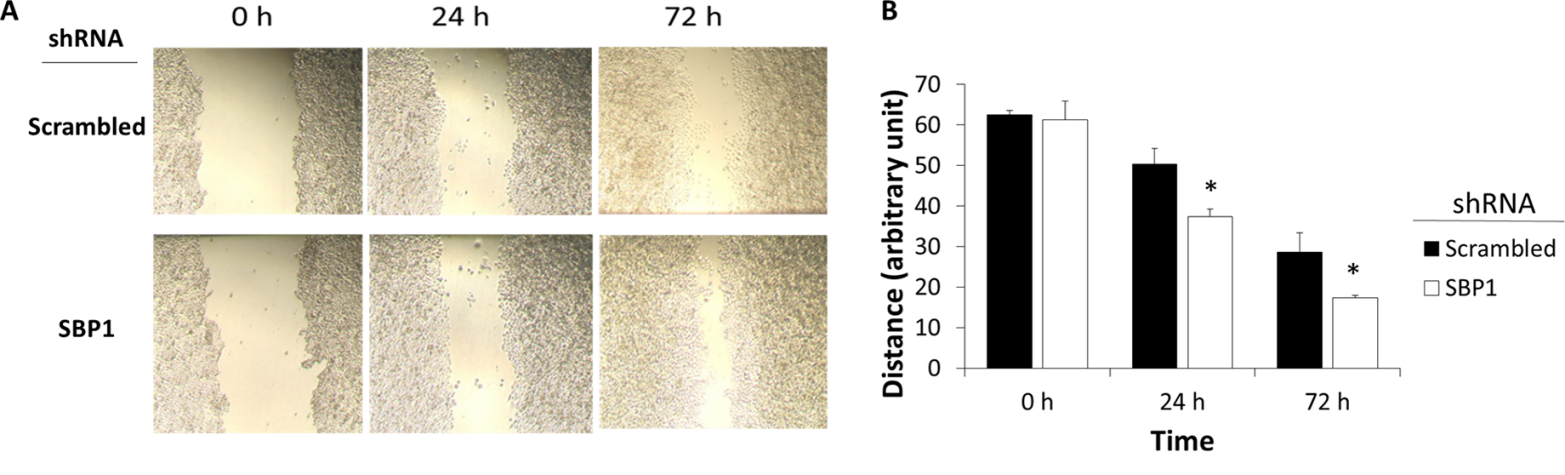
Knockdown of SBP1 promotes HeLa cell migration. (A) Cells cultured in DMEM supplemented with 0.2% FBS were made the wound on cell culture dishes and visualized at 0–72 h. (B) The wound distance was semi-quantified by randomly selecting the shortest distance between the two patches of cells. Values are means ± SEM (n = 3).*, *p*< 0.05, compared to scrambled shRNA cells.

We did not noticeably observe apparent morphology changes between SBP1 and scrambled shRNA cells. Because the doubling time of HeLa cells was approximately 24 h, cell migration was monitored 24 and 72 h after a scratch was made. The SBP1 shRNA HeLa cells migrated faster than the scrambled cells during the 72-h time course (Fig 4). Results from SRB assays showed that there was a small (5.8%) yet statistically significant difference (*p<*0.05) in proliferation between SBP1 (3.10 ± 0.05) and scrambled (2.92 ± 0.05) shRNA HeLa cells 3 days after seeding the cells without any treatment. This suggests that there was little off-target effect of SBP1 shRNA knockdown and the observed promotion of migration in wounded SBP1 shRNA cells was only minimally attributed to the intrinsic difference in cell proliferation.

## Discussion

The two major selenium chemoprevention clinical studies at supranutritional levels of oral supplementation yield mixed results [28, 29], but such seemingly discrepancy appears to be compromised by a couple of confounding factors (for details, see [30]). Loss of SBP1 is known to aggravate cancer invasion and serve as an indicator of poor prognosis at late stage of carcinogenesis [10]. One of the key observations made in this study is that SBP1-deficient HeLa cells show reduced selenium retention when treated with selenomethionine at a dose relevant to supranutritional levels of selenium. Such doses can induce oxidative stress in cancer cells [22]. Thus, we speculate that decreased oxidative stress may contribute to increased chemoresistance in SBP1-deficient cancer cells. DNA-damaging agents have long been employed for cancer chemotherapy [28]. In the current research, we have shown that SBP1 deficiency confers resistance of HeLa cells to clastogens. Hydrogen peroxide at concentrations below 10 μM typically enhances survival and stimulates proliferation in cultured cells, whereas higher concentrations induce DNA damage [29]. Camptothecin, a cytotoxic quinoline alkaloid, binds topoisomerase I and DNA to form a complex that interferes replication and other DNA metabolism events, resulting in activation of DNA damage and apoptotic responses. Camptothecin is also known to induce lipid peroxidation in rats [30]. Paraquat is a pro-oxidant that facilitates the transfer of electron to oxygen for superoxide formation [31]. Although awaiting experimental verification, our observation that knockdown of SBP1 desensitizes HeLa cells to these three clastogens indicates that SBP1 may retain supplemental selenium and limit proliferation through promotion of oxidative stress and genome instability.

Lowered intracellular selenium content may also confer SBP1-deficient HeLa cells resistance to clastogens. Selenium compounds have been shown to protect against various stages of carcinogenesis [32]. SBP1 possibly binds selenium via a selenosulfide bond (perselenide) at cysteine-57 [4, 5]. SBP1-deficient HeLa cells retain less selenium (Fig 3), a scenario probably in favor of cancer cell survival. However, because intracellular selenium contents increase linearly in both SBP1 and scrambled shRNA cells and the rate diverts when selenomethionine concentrations are > 2 μM, this suggests that SBP1 plays such a critical role in retaining selenium only when selenium doses are high. Furthermore, because selenomethionine at 1 and 2 μM also induces SBP1 expression, selenium at such lower doses may be retained in a non-specific manner such as replacement of sulfur in sulfur-containing amino acids. Similarly, mythylselenocysteine can directly increase SBP1 expression [13]. Consistent with this report, we show that selenomethionine treatment increases SBP1 protein expression in HeLa cells. On the other hand, supplementation of sodium selenite (up to 250 nM) does not induce SBP1 expression in MCF-7 cells while such doses suppress SBP1 expression under a GPX1-overexpressing background [17]. This suggests that SBP1 expression is induced by supplemental selenium only at high doses, and there is a competition on using available selenium between SBP1 and GPX1 [17]. Although our results suggest that selenium administration may be considered for cancer patients with reduced SBP1 expression, it would be necessary first to elucidate the temporal kinetics of selenium retention by SBP1 and determine whether such SBP1 function exists in other selenium species and cell types.

Reductive stress due to GPX1 overexpression can result in adverse health consequences such as type-2 diabetes [33]. Furthermore, GPX1 level is closely related with cancer risks [15, 34–36]. However, SBP1 seems to have antagonistic effects against GPX1, as reciprocal interactions between SBP1 and GPX1 have been reported [9, 17]. SBP1 and GPX1 can co-localize in cells under oxidative stress [9]. As a selenoprotein sensitive to body selenium fluctuation, GPX1 likely interacts with SBP1 in the response of cancer cells to supplemental selenium or changes in redox status.

ROS can be produced intrinsically from mitochondria respiration and redox enzymes or extrinsically from environmental pollution such as the herbicide paraquat [37]. Low or physiological levels of ROS are necessary for normal signalling and metabolic homeostasis, whereas ROS imbalance is associated with many pathophysiological events such as carcinogenesis [38]. ROS can induce various forms of DNA damage [39]. Knockdown of SBP1 in HeLa cells decreases ROS levels in the absence or presence of hydrogen peroxide treatment. Such suppressed oxidative stress may be attributed to GPX1, as SBP1 is known to negatively regulate GPX enzymatic activity [9, 15, 17, 18] and we show SBP1 knockdown increases GPX1 protein expression (Fig 1B). GPX1 accounts for the majority of hepatic selenium and mediates body selenium to protect against paraquat toxicity in mice [40]. Furthermore, GPX1 overexpression protects cells from UV-induced DNA damage [41]. It is of future interest to study the interplay between ROS, SBP1 and antioxidant selenoproteins during carcinogenesis.

A common problem in cancer therapy is chemoresistance. Cancer cells show resistance to a variety of structurally or functionally distinct agents, especially when relapse happens. Previous reports have shown that knockdown of SBP1 accelerates proliferation and migration in human SMMC7721 hepatic and HCT116 colorectal cancer cells [9, 42]. Consistent with these data, our current results in HeLa cervical cells expand the list of cancer cells exhibiting an inhibitory role of SBP1 in cancer migration, suggesting that this barrier role of SBP1 in late stage carcinogenesis is general but not specific to certain cancers. Furthermore, our finding of reduced oxidative stress and cellular selenium content in SBP1 shRNA cells suggests plausible reason why SBP1 can inhibit cancer cell growth and proliferation. In conclusion, we provide evidence that suggests that decreased ROS formation under SBP1 deficiency can promote migration and chemoresistance. In addition to targeting SBP1, cancer-specific delivery of selenium compounds or pro-oxidants may be of clinical potential as a complementary approach to current strategy treating cancer.
